# Fluorescence intensity monitors as intensity and beam-position diagnostics for X-ray free-electron lasers

**DOI:** 10.1107/S1600577519001802

**Published:** 2019-02-22

**Authors:** Philip Heimann, Alexander Reid, Yiping Feng, David Fritz

**Affiliations:** a Linac Coherent Light Source, SLAC National Accelerator Laboratory, 2575 Sand Hill Road, Menlo Park, CA 94025, USA

**Keywords:** X-ray intensity monitor, diagnostics, X-ray free-electron laser

## Abstract

For LCLS-II, a fluorescence intensity monitor for the non-invasive, pulse-by-pulse normalization of experiments has been developed. A prototype diagnostic was constructed with a microchannel plate assembly and two photodiodes. The diagnostic was then installed in the LCLS SXR instrument Kirkpatrick–Baez mirror chamber with the detectors located above the vertically reflecting mirror. The linearity, noise and position sensitivity of the detectors have been characterized. The photodiode responsivity is suitable for high pulse energies. The microchannel plate detector shows sufficient responsivity over a wide range of pulse energies. The relative signal from the two photodiodes provides a sensitive measure of the X-ray beam position. The fluorescence intensity monitor provides intensity normalization while being compatible with high incident power, a 0.93 MHz repetition rate and ultra-high vacuum.

## Abstract 2

A fluorescence intensity monitor has been developed for the non-invasive, pulse-by-pulse normalization of LCLS-II experiments. The linearity, noise and position sensitivity of the detectors have been characterized.

## 1. Introduction   

The LCLS-II project is constructing a 4 GeV superconducting accelerator for the production of X-ray free-electron laser radiation. LCLS-II will generate X-rays at repetition rates up to 0.93 MHz and cover a photon energy range from 250 eV to 5000 eV. The average power is expected to exceed 200 W over the majority of the energy range. LCLS-II commissioning is scheduled to begin in 2020. Three X-ray instruments, 1.1, 1.2 and 2.2, are being designed for LCLS-II. Instrument 1.1 delivers high-flux soft X-rays to multiple endstations including a dynamic-reaction microscope. Instrument 1.2 covers the tender X-ray photon energy range up to 5000 eV and allows X-ray beams from both the soft X-ray and hard X-ray undulators to be combined at the sample. Instrument 2.2 delivers moderate and high-resolution monochromatic X-rays for techniques such as resonant inelastic X-ray scattering. Providing SASE bandwidth at some locations and high resolution at another, these instruments will deliver a variety of pulse energies to experiments.

The fluorescence intensity monitor (FIM) is being developed as a non-invasive intensity monitor for the LCLS-II X-ray instruments. The goal is to achieve a signal-to-noise ratio of 100:1 for individual X-ray pulses. A large dynamic range is required to meet the requirements of LCLS-II X-ray instruments. The anticipated pulse energies range from 0.2 µJ at Instrument 2.2, operating for high-resolution momentum resolved resonant inelastic scattering experiments, to 12 mJ at Instrument 1.1, operating for strong-field experiments. The highest pulse energies are generated by the 120 Hz copper LINAC electron beam being delivered into the soft X-ray undulator. The diagnostic should provide pulse-by-pulse data at the LCLS-II maximum repetition rate, 0.93 MHz. The FIM should also be compatible with the full LCLS-II photon energy range, 250 eV to 5000 eV.

A number of intensity diagnostics have been developed for X-ray FELs such as the gas monitor detector (GMD) and the intensity position monitor (IPM). The GMD uses photoionization from rare gas atoms (Tiedtke *et al.*, 2008 ▸, 2014 ▸). It is non-invasive because of the low gas density, and provides absolute intensities from a calibration at the Radiometry Laboratory of the PTB at BESSY. However, a considerable length along the X-ray path is needed for the GMD and the associated differential pumping. In addition, the GMD requires complex controls. The IPM detects back-scattered X-rays from thin, partially transmissive silicon nitride or diamond foils (Feng *et al.*, 2011 ▸; Tono *et al.*, 2011 ▸). With a target thickness chosen according to the photon energy and photoabsorption cross-section, the transmission of the IPM can be high, and their responsivity can be calibrated (Kato *et al.*, 2012 ▸). But at a high average power, the cooling of the thin targets is challenging.

The concept of the FIM is to place detectors near an X-ray mirror surface in order to collect X-ray fluorescence and, in some cases, photoelectrons. This method provides a non-invasive measurement because there is no foil in the beam path. Furthermore, it does not occupy valuable space along the X-ray transport. High incident powers are possible because the X-rays are primarily reflected by the mirror, which will be water-cooled. Since photodiodes and microchannel plate detectors can have a response time of a few nanoseconds, the diagnostic is compatible with the maximum LCLS-II repetition rate. Relative X-ray pulse energies will be determined. It is planned for absolute X-ray intensities to be measured by gas detectors in the front-end enclosure (Hau-Riege *et al.*, 2010 ▸) and by power meters in the X-ray instruments (Heimann *et al.*, 2018 ▸).

## 2. Fluorescence intensity monitor prototype   

**Figure 1:**
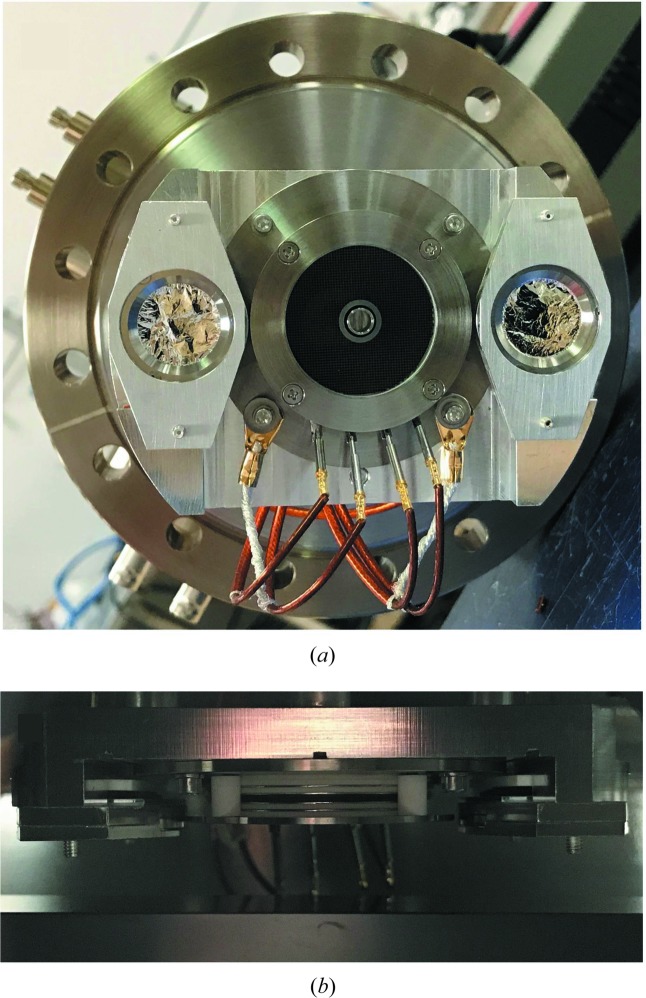
(*a*) Photograph of the assembled FIM prototype. (*b*) Photograph of the FIM prototype installed above the LCLS SXR vertical KB mirror.

A prototype FIM was fabricated including a microchannel plate detector and two photodiodes. The Hamamatsu model F2223-21SH microchannel plate detector has a grid and two channel plates and was available from a previous diagnostic. The photodiodes, Optodiode model AXUV63HS-1, were selected for their rise time of 2 ns. The photodiodes are positioned behind 100 nm-thick Al filters to supress electrons. Fig. 1 ▸(*a*) shows a photograph of the assembled FIM prototype mounted on a 6 inch diameter flange. As seen in Fig. 1 ▸(*b*), the FIM prototype was installed above the vertically reflecting Kirkpatrick–Baez (KB) mirror (Heimann *et al.*, 2011 ▸) in the LCLS Soft X-ray Materials Science (SXR) Instrument (Dakovski *et al.*, 2015 ▸). To ensure that the X-ray beam would pass the diagnostic, the closest distance from the diagnostic assembly to the mirror surface was kept at 10 mm. In order to preserve the cleanliness of the mirror surface, the diagnostic should not negatively affect the mirror chamber vacuum. Bakeouts as well as residual gas analysis (RGA) scans were performed on the individual components of the FIM prototype. The RGA results met the LCLS vacuum specification for beamline components, which requires that at room temperature the sum of the partial pressures of all the peaks above 44 AMU must be less than 1 × 10^−11^ Torr and the maximum single partial pressure above 44 AMU must be less than 5 × 10^−12^ Torr. No bakeout of the mirror chamber was performed. After 12 days, the SXR KB mirror chamber reached a pressure of 2 × 10^−8^ Torr, and after two months the pressure was 3 × 10^−9^ Torr.

## 3. Characterization at the LCLS SXR instrument   

Measurements were performed at the LCLS SXR instrument to characterize the performance of the FIM prototype. The photodiode and MCP detector signals were read pulse-by-pulse into an Acqiris U1065A-DC282 digitizer using a 20 MHz filter and then stored by the LCLS data acquisition system. The photodiodes were given a reverse bias of 9 V while the MCP detector voltages were adjusted for the desired gain. The pulses have a 28 ns full width at half-maximum (FWHM) for the photodiodes and 24 ns FWHM for the MCP detector limited by the filter. These observed pulse widths are compatible with the maximum LCLS-II repetition rate of 0.93 MHz.

**Figure 2:**
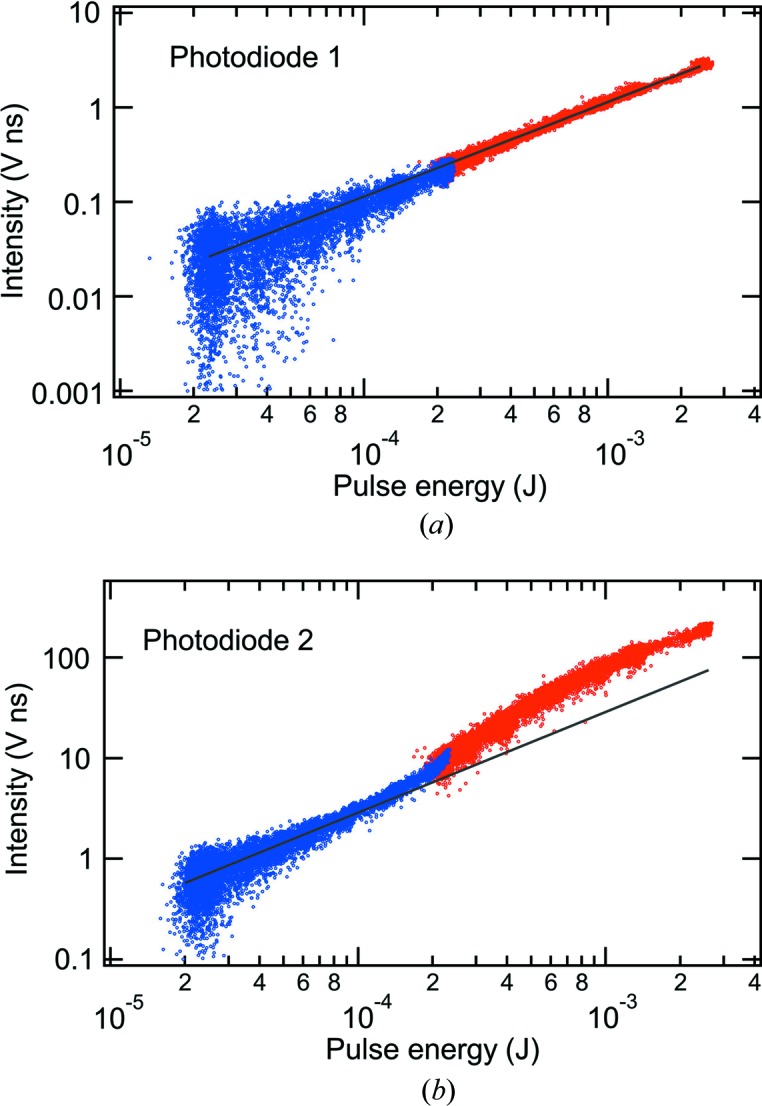
The responsivity of photodiodes 1 and 2 compared with the X-ray pulse energy calculated from the LCLS upstream gas detector and gas attenuator at a photon energy of 1540 eV. The solid lines represent linear fits: *y* = *c* *x*. The colors correspond to individual runs, during which the gas attenuation is increased by a factor of ten.

**Figure 3:**
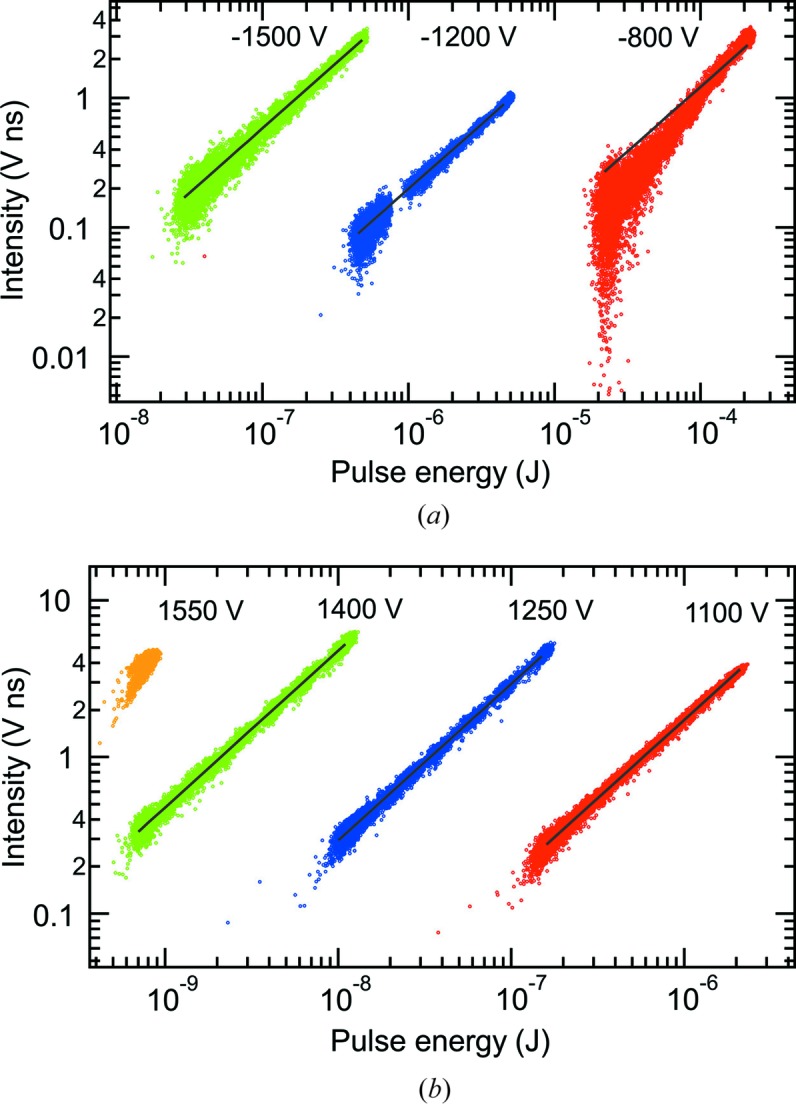
The responsivity of the MCP detector compared with the X-ray pulse energy calculated from the LCLS upstream gas detector and gas attenuator. The solid lines represent linear fits: *y* = *cx*. (*a*) At a photon energy of 1540 eV, a negative bias was applied with the MCP input voltage varied from −800 V to −1500 V. (*b*) At a photon energy of 1480 eV, a positive bias was applied with the anode voltage varied from 1100 V to 1550 V.

The linearity of the detectors was tested by varying the X-ray pulse energy using the gas attenuator (Ryutov *et al.*, 2009 ▸) in the LCLS front-end enclosure. Fig. 2 ▸ displays the signals of photodiodes 1 and 2 *versus* the pulse energy derived from the upstream gas detector (Hau-Riege *et al.*, 2008 ▸) and the calculated gas attenuation. Fig. 3 ▸ similarly shows the MCP detector signal *versus* the pulse energy from the upstream gas detector and the gas attenuation. Each point represents a single X-ray pulse event. The photodiode and MCP detector intensities were calculated by subtracting the background and then integrating over the pulse. The SXR instrument was operated in non-monochromatic mode. Results from the commissioning of the SXR instrument (Tiedtke *et al.*, 2014 ▸) predict that under these conditions the X-ray pulse energy downstream of the KB mirror chamber is 34% of the value in the front-end enclosure. An empirical correction has been made to the gas attenuation from the Beer–Lambert law. The attenuation, *I*/*I*_0_, is calculated from the nitro­gen density, *n*, in the attenuator,
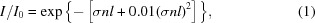
where σ is the photoabsorption cross-section and *l* is the attenuator length. Increased attenuation can be caused by additional pressure along the X-ray beam path in the attenuator differential pumping sections. This correction to the gas attenuation is consistent with measurements where the power meters and the SXR gas monitor detector were correlated with the pulse energy from the gas attenuator and gas detectors.

In Fig. 2 ▸, photodiode measurements were performed at a photon energy of 1540 eV and with LCLS pulse energies ranging from 20 µJ to 2.6 mJ. The photodiode 2 signal is higher than that of photodiode 1 because the X-ray beam footprint is almost centered below photodiode 2 and, as a consequence, photodiode 1 is sampling a tail of the beam footprint. At this photon energy, the X-ray beam FWHM projected upon the mirror is less than the separation between the photodiodes. The photodiode 1 signal is observed to be linear within the noise, as seen by the fit *y* = *cx* shown by line in Fig. 2 ▸(*a*). For photodiode 2, at pulse energies above 100 µJ, curvature is observed in the response. At high pulse energies, photodiodes offset from the center of the X-ray beam footprint should be used for the intensity normalization. The photodiodes demonstrate a dynamic range of about one order of magnitude.

In Fig. 3 ▸, the MCP detector is operated in two modes: with negative and positive bias. In the case of negative bias, electrons are suppressed by the negative potential on the grid, which is placed 500 V more negative than the MCP input, *i.e.* between −1300 V and −2000 V. Photoemission is not completely suppressed in the case of a −1300 mesh voltage. With positive bias, the grid and MCP input are given small positive voltages, and both electrons and X-rays are detected. By comparing the MCP detector signal with positive and negative bias, it is estimated that the number of electrons exceeds the number of X-rays by about a factor of 100. The bias voltages, in particular the voltage difference across the MCPs, allow the gain to be adjusted according to the pulse energy. For negative bias, the MCP voltage difference is given by the MCP input voltage plus 50 V. For positive bias, the MCP voltage difference is equal to the anode voltage minus 295 V. With negative bias, measurements were performed at a photon energy of 1540 eV and with LCLS pulse energies ranging from 30 nJ to 230 µJ. Here, the MCP detector signal is seen to be linear within the noise except for the case of −800 V MCP input voltage, where the intensity increases faster than the linear fit. With positive bias, measurements were performed at a photon energy of 1480 eV and with LCLS pulse energies ranging from 0.7 nJ to 2 µJ. Over this range of pulse energies, the MCP detector signal is seen to be linear within the noise variations. The MCP negative bias mode covers the pulse energy range from 2 µJ to 100 µJ, where the positive bias mode is no longer linear. The MCP positive bias mode has superior performance below 2 µJ. The MCP detector demonstrates a dynamic range of four orders of magnitude. From the LCLS-II Instrument 2.2 requirements, a sensitivity at 0.2 µJ pulse energy is needed, which is clearly met by the MCP detector.

The photodiode and MCP detector noise was evaluated by comparison with an Andor Newton CCD camera using selectable filters to maintain single-photon detection. The noise contribution of the CCD camera in the full-frame readout mode may be derived from Poisson statistics for a maximum *N* of 5 × 10^5^ detected X-rays, *i.e.* *N* ^1/2^/*N* = 1.4 × 10^−3^. The noise measurements were performed at 800 eV. The standard deviation σ is calculated from the residual of a linear fit of the photodiode or MCP detector intensity *versus* the CCD camera intensity integrated over the exposed area. The noise results (σ divided by the average intensity) are listed in Table 1 ▸. At higher pulse energies, the photodiode noise is 4–5% of the intensity, and it increases slowly with decreasing pulse energy. Here the MCP detector was operated with positive bias. The MCP detector noise is 2–3% and insensitive to the pulse energy. The main noise contributions are electronic noise, the pulse-height distribution from microchannel plates (Matsuura *et al.*, 1985 ▸) and Poisson statistics. The weak variation with pulse energy suggests that Poisson statistics is not the dominant noise contributor.

**Figure 4:**
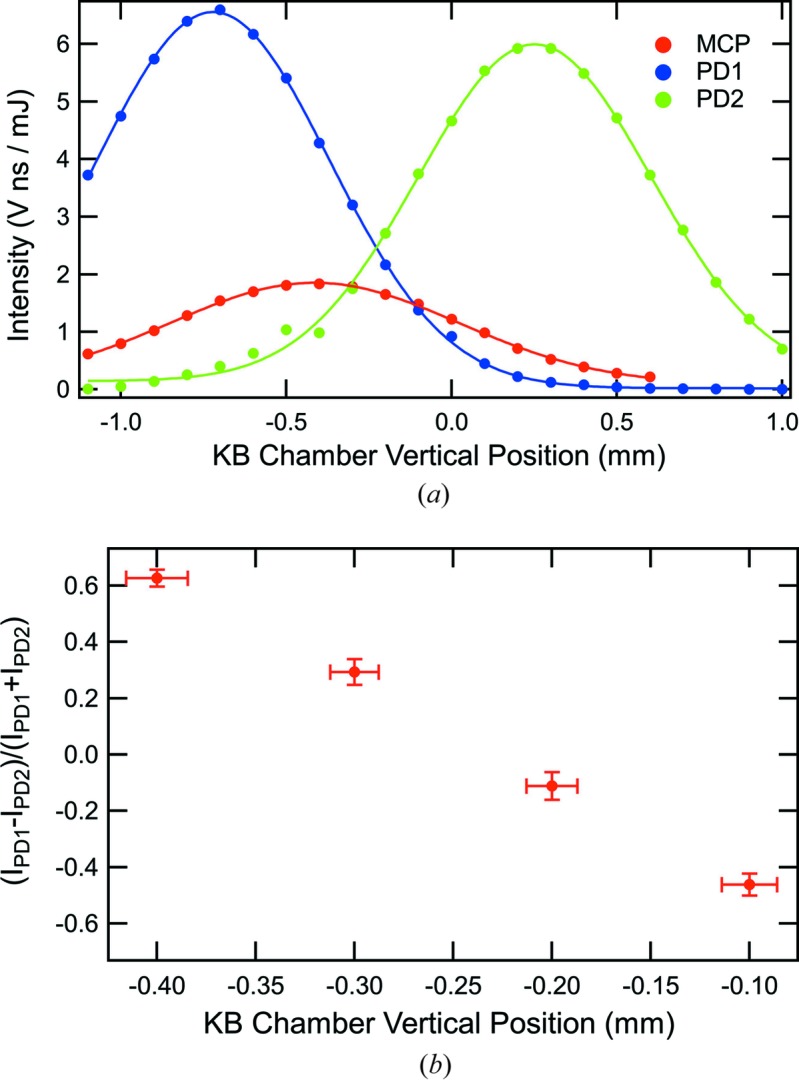
(*a*) At 800 eV photon energy, the intensities of photodiodes 1 and 2 and the MCP detector as the KB chamber is translated vertically. (*b*) Derived from the photodiode intensities in Fig. 4 ▸(*a*), *f*(*y*) from equation (2) is shown. The error bars are calculated from the σ/*I* values in Table 1 ▸ at 8.0 × 10^−4^ J pulse energy.

To investigate the sensitivity of the FIM diagnostic to the X-ray beam position, the vertical position of the KB mirror chamber was scanned, equivalent to translating the X-ray beam, while monitoring photodiode 1 and 2 and MCP detector intensities. Fig. 4 ▸(*a*) displays such a scan at 800 eV photon energy. Here, the detector intensities were calculated by subtracting the background, integrating over the pulse and then normalizing to the pulse energy from the upstream gas detector. Each point represents the mean of a run, typically ∼7000 X-ray pulse events. The intensities from all three detectors vary with the vertical height of the chamber. A discontinuity in the photodiode 2 curve at −0.5 mm correlates with a change in the digitizer voltage range. The vertical width of the photodiode 1 and 2 intensity curves is 0.8 mm, which is consistent with the image observed on an yttrium–aluminium garnet (YAG) screen at the SXR single-pulse shutter. The 0.8 mm vertical width corresponds to an X-ray beam footprint along the mirror of 59 mm. The width of the MCP detector intensity curve is broader than that of the photodiodes because of the respective active diameters, 27 mm for the MCP detector compared with 9 mm for the photodiodes. The FIM detectors are sensitive to the X-ray beam position.

The sensitivity of the diagnostic to the X-ray beam position was evaluated by considering the variation of the photodiode signals with KB mirror chamber position as well as the noise σ when averaging over a number of X-ray pulse events. As in the measurement of the detector noise listed in Table 1 ▸, the photodiode intensities were compared with the CCD camera. To consider averaging, the CCD camera was operated in full vertical bin mode, which permitted 120 Hz data acquisition. To use the intensities from the two photodiodes as a vertical position encoder, one may calculate a function *f*(*y*),
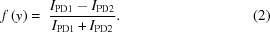
Using the photodiode intensities in Fig. 4 ▸(*a*), Fig. 4 ▸(*b*) displays *f*(*y*) at KB chamber vertical positions where the center of the X-ray beam footprint is located between photodiodes 1 and 2. The vertical error bars for *f*(*y*) are calculated from the photodiode 1 and 2 σ/*I* values appearing in Table 1 ▸ at 8.0 × 10^−4^ J pulse energy. The horizontal error bars represent a conversion of the noise in *f*(*y*) into a single-shot uncertainty in the X-ray beam position. The noise σ/*I* improves with averaging from 0.05 for single X-ray events to 0.008 when summing 100 X-ray pulse events. For averaging 100 X-ray pulse events, an uncertainty of 2 µm in the vertical beam position is derived. In conclusion, it is possible to use the FIM for measuring the X-ray beam position and for feedback correction of thermal drift. To obtain both horizontal and vertical positions, FIMs would need to be implemented for both the horizontally and vertically reflecting KB mirrors.

The present characterization of the FIM was constrained by the photon energy range of the LCLS SXR instrument, 280 eV to 2000 eV (Dakovski *et al.*, 2015 ▸). An FIM could be implemented in a similar manner for higher photon energy X-rays with some differences in sensitivity. At higher photon energies, the fluorescence yield will be increased in the case of higher *Z* coatings. The microchannel plate efficiency varies with photon energy, having a minimum at 4–5 keV (Yamaguchi *et al.*, 1989 ▸).

## 4. Summary   

The fluorescence intensity monitor has been developed as a pulse-by-pulse relative intensity diagnostic for the LCLS-II X-ray instruments. Pulse energy measurements at the maximum LCLS-II repetition rate are feasible. The photodiodes show a suitable responsivity at high pulse energies. The MCP detector demonstrates high responsivity for low pulse energies even in the nJ range. Over appropriate pulse ranges, linearity is observed for both the photodiodes and MCP after an empirical correction to the gas attenuation. Noise levels of 4–5% for the photodiodes and 2–3% for the MCP detector have been demonstrated. The intensity from multiple detectors can be used to determine the X-ray beam position, which may provide feedback correction of thermal drift. For the LCLS-II X-ray instruments, there are plans to install FIMs in the KB mirror chambers.

## Figures and Tables

**Table 1:** Evaluation of the noise of the photodiodes and MCP detector

Pulse energy (J)	Photodiode 1 (σ/*I*)	Photodiode 2 (σ/*I*)	MCP (σ/*I*)
8.0 × 10^−4^	0.044	0.055	
1.2 × 10^−4^	0.058		
1.5 × 10^−5^			0.024
1.1 × 10^−6^			0.027
1.2 × 10^−7^			0.028
